# Exercise-induced bronchospasm: A case study in a nonasthmatic patient

**DOI:** 10.1111/j.1745-7599.2011.00691.x

**Published:** 2012-01

**Authors:** Mary Lou Hayden, Stuart W Stoloff, Gene L Colice, Nancy K Ostrom, Nemr S Eid, Jonathan P Parsons

**Affiliations:** 1 University of VirginiaCharlottesville, Virginia; 2 Department of Family and Community Medicine, School of Medicine, University of Nevada RenoNevada; 3 Pulmonary, Critical Care and Respiratory Services, Washington Hospital Center, Silver Spring, Maryland, and The George Washington University School of MedicineWashington, DC; 4 Allergy & Asthma Medical Group and Research CenterSan Diego, California; 5 Pediatric Pulmonary Medicine, and Cystic Fibrosis Center, University of LouisvilleLouisville, Kentucky; 6 Pulmonary, Allergy, Critical Care, and Sleep Medicine, Ohio State University Asthma CenterColumbus, Ohio

**Keywords:** Asthma, bronchospasm, exercise, pharmacotherapy, health promotion

## Abstract

**Purpose:**

To provide an overview of the clinical presentation, diagnosis, and management of exercise-induced bronchospasm (EIB) without underlying asthma.

**Data sources:**

Case presentation and review of the EIB Landmark Survey.

**Conclusions:**

EIB is a common and well-described occurrence in patients with asthma, as well as in patients with no overt respiratory condition. Treatment with a short-acting beta-agonist before starting exercise is effective, yet this treatment approach is underutilized in the majority of patients with asthma.

**Implications for practice:**

This case highlights the implications of undermanaged EIB and the disconnect between healthcare provider recommendations and the beliefs and behaviors in patients with EIB. Inhaled short-acting beta-agonists can attenuate EIB in 80%–95% of patients and are effective during 2–3 h of exercise. Patients with a compromised level of physical activity because of EIB who do not respond to conventional treatment strategies should be referred to a respiratory specialist for diagnostic evaluation and confirmation of underlying asthma. Nurse practitioners should remain vigilant to identify untreated EIB and ensure that affected patients understand the condition and appropriate treatment options.

## Overview

Up to 90% of patients with asthma are reported to have exercise-induced asthma (EIA) or exercise-induced bronchospasm (EIB; McFadden & Gilbert, 1994), and as many as 12%–15% of patients without asthma may develop EIB (Gotshall, 2002), usually during or within 20 min of finishing moderate to vigorous exercise. Rather than seeking medical diagnosis and management, individuals with asthma or EIB may decide not to participate in exercise, leaving them at higher risk for other conditions, such as obesity, which may impact their overall health and lifestyle habits (Dogra, Baker, & Ardern, 2009). The widespread incidence of EIB, particularly in patients with asthma but also in those with no overt respiratory condition, suggests that healthcare professionals will encounter such patients regularly in their day-to-day practice.

Although the pathogenesis of EIB is not fully elucidated, it is probably caused by exercise-induced hyperventilation and corresponding changes in airway physiology (Weiler et al., 2010). A history of cough, shortness of breath, chest pain or tightness, wheezing, or endurance problems during exercise suggests EIB. However, use of history alone has been shown to both underdiagnose and overdiagnose the problem (McKenzie et al., 2002; Tan & Spector, 2002). An exercise challenge is often used to establish the diagnosis, although additional indirect and direct bronchoprovocation tests can also be used to confirm diagnosis (Parsons, 2010; Stack & Hakemi, 2011). Typically, a 10%–15% decrease in peak expiratory flow or forced expiratory volume in 1 s (FEV_1_) after an indirect or direct bronchoprovocation test is compatible with EIB (U.S. Department of Health and Human Services, 2007; Weiler et al., 2010).

The recent EIB Landmark Survey (http://www.eiblandmarksurvey.com, 2009) was a multisurvey initiative that reported on subjects with and without a diagnosis of asthma with an objective of better understanding the impact of exercise-related respiratory symptoms. As part of this initiative, a general public survey (*N*= 1000) included individuals who have no diagnosis of asthma (86%) and those who have a diagnosis of asthma or who currently take asthma medications (14%). Result highlights included the fact that 5% were diagnosed as having EIA or EIB (29% of the asthma population and 2% of the nonasthma population), while another 24% of the general adult population reported experiencing at least one of six exercise-related respiratory symptoms during or immediately after exercising: shortness of breath, wheezing, coughing, difficulty taking a deep breath, noisy breathing, or chest tightness. There was a weak correlation between exercise-related respiratory symptoms and diagnosis of EIA or EIB (*r*= .045, *p*= .077). The proportion of adults diagnosed with EIA or EIB was 7% for those who experienced 1 or 2 of these EIB symptoms. The diagnosis of EIA or EIB increased to 11% with three symptoms, 19% with four symptoms, 24% with five symptoms, and 35% with all six symptoms. However, the majority (65%) of adults who experienced respiratory symptoms during or shortly after exercise or physical exertion had never been diagnosed with EIA or EIB.

Treatment for EIB is well established (U.S. Department of Health and Human Services, 2007). Prophylactic use of a short-acting beta-2 agonist (SABA) inhaler, such as albuterol, levalbuterol, or pirbuterol, before starting moderate exercise is highly effective in 80%–95% of patients with EIB (Milgrom & Taussig, 1999; U.S. Department of Health and Human Services, 2007). However, despite this widely accepted approach to treatment, the EIB Landmark Survey reported that less than 25% of asthma patients with exercise-related respiratory symptoms use quick-relief medicine before exercising. Thus, many patients fail to effectively manage their EIB. In the EIB Landmark Survey, 96% of healthcare providers, but only 57% of patients, agreed that quick-relief medications should be taken before exercise to reduce the chances of an asthma attack.

Next, we present the case of a patient with EIB without underlying asthma, illustrating the impact that this condition can have when not managed effectively. All possible identifying information has been excluded.

## Case study

A 34-year-old female visits her nurse practitioner (NP) with complaints of fatigue and lethargy. She is overweight, with a body mass index of 28, and has a history of depression, for which she has been taking a selective serotonin reuptake inhibitor since the birth of her second child 5 years ago. Her blood pressure is 120/80 mmHg, and routine laboratory tests reveal elevated serum triglyceride and low high-density lipoprotein values. She has no other remarkable clinical history, including no history of asthma, recurrent bronchitis, or tobacco use. The patient denies cough, chest pain, palpitations, peripheral edema, and nocturnal awakening with respiratory symptoms. Additional questioning reveals that the patient has avoided recreational activity since adolescence because it often left her feeling out of breath, with mild wheezing, unable to keep up with her peers. She has gained 40 lb since the birth of her second child, which she feels has “slowed her down.”

A review of the patient's medical history reveals that until recently she has led a largely sedentary home life and has only recently begun to participate in recreational exercise because of her commitment as a single mother to her two daughters. She has also started taking brief walks with her children around the track, which results in shortness of breath within 5–8 min of beginning exercise. She is not accustomed to this new level of physical activity and is experiencing shortness of breath, chest tightness, and wheezing on exhalation no more than twice a week, which resolves if she rests or drinks coffee.

## Physical examination

Head, eye, ear, nose, and throat examinations were unremarkable, and chest examination showed normal excursion and was clear upon auscultation. Heart sounds showed regular rate and rhythm, with no S3 or S4 murmur or gallop. No signs of peripheral edema were present, and the skin was normal.

## Differential diagnosis

Common differential diagnoses when a young adult patient complains of shortness of breath include asthma and other pulmonary diseases, cardiovascular disease, vocal cord dysfunction, obesity, poor conditioning, and dyspnea as a result of anemia.

## Diagnostic testing and treatment

**Figure 1:**
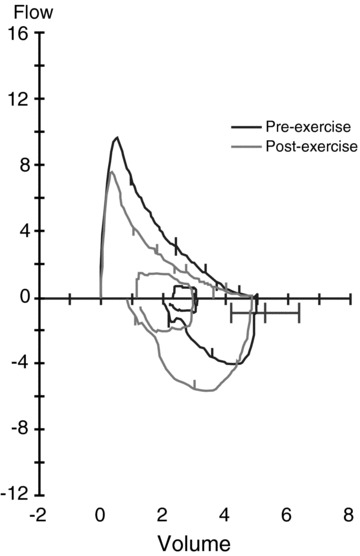
Spirometry results showing pre- and post-exercise challenge.

The patient was referred for further diagnostic testing. Chest x-ray was normal and complete blood count within normal limits. An exercise challenge revealed oxygen saturation of 99% at rest and 98% after a 6-min walk. Pulmonary function tests were ordered and revealed normal flow volume loop, normal lung volumes, and FEV_1_ (90% of predicted). While her baseline spirometry reading was normal, a second spirometry after exercise challenge revealed a significant decrease (15%) in FEV_1_(Figure 1). The patient's NP makes a presumptive diagnosis of EIB.

Patients with suspected EIB should be evaluated with a detailed history and physical examination (including ear, nose, and throat and cardiac and chest examinations) and lung function measurements before and after SABA administration. A decrease of at least 10% in FEV_1_ after exercise is sufficient to make the diagnosis of EIB, especially if symptoms accompany the drop in FEV_1_ (U.S. Department of Health and Human Services, 2007; Weiler et al., 2010). A diagnosis of reversible airway obstruction is made if FEV_1_ improves by 12% (and 200 mL) at 15 min after SABA administration (Crapo et al., 2000). Often, EIB can be prevented by use of a SABA inhaled within 15 min before exercise; some authors suggest that response to this therapy can serve as a basis for a diagnosis of EIB. In many patients, bronchial hyperresponsiveness should be evaluated with an exercise challenge or a surrogate challenge (e.g., with cold air hyperventilation, methacholine, adenosine 5-monophosphate, or mannitol challenge).

For patients in whom spirometric testing does not definitively diagnose EIB, formal bronchoprovocation testing may be performed. Given greater availability, an exercise challenge is more typically used. Exercise and eucapneic voluntary hyperventilation (EVH) testing are examples of indirect airway challenges that act by causing the release of endogenous mediators of airway smooth muscle contraction. In athletes, EVH testing, performed in a specialized pulmonary function laboratory, is the most sensitive test for diagnosing EIB. In patients with risk of coronary heart disease, cardiac monitoring and immediate availability of resuscitation resources are required. Thus, healthcare practitioners often seek other types of challenges for these adults. A mannitol bronchial challenge test (Aridol™) has recently been approved in the United States for the assessment of bronchial hyperresponsiveness in patients 6 years of age or older and has been used to diagnose EIB. Responses to challenges with other indirect-acting stimuli (adenosine 5-monophosphate, hypertonic saline, and cold dry air) tend to correlate with each other, and several have been studied as surrogate challenge agents for EIB. This is in contrast to the action of methacholine and histamine, which provoke bronchoconstriction by direct action on airway smooth muscle (Weiler et al., 2010).

Treatment for EIB is well established (Millward, Tanner, & Brown, 2010; Stack & Hakemi, 2011). The National Asthma Education and Prevention Program Expert Panel (EPR-3) convened by the National Heart, Lung, and Blood Institute reviewed the evidence for the treatment and management of EIB. Shortly before exercise, a SABA administered for the prevention of EIB may be helpful for 2–3 h. Two puffs of a SABA, such as albuterol, levalbuterol, or pirbuterol, before exercise are effective in more than 80% of cases. Long-acting beta-2 agonists (LABAs) have a longer duration of action and can be protective for up to 12 h. However, when LABAs are administered on a daily basis, there is some shortening of the duration of protection, even in patients using inhaled corticosteroids. The EPR-3 guidelines recommend that frequent and long-term use of LABAs for EIB should be discouraged. Such use may disguise poorly controlled persistent asthma, which should be managed with a daily anti-inflammatory. The Food and Drug Administration recently required a label change for both approved LABAs in the United States: use without a long-term controller, such as an inhaled steroid, is contraindicated. Leukotriene receptor antagonists can attenuate EIB in up to 50% of patients, but onset of action is generally hours after administration. Given that the patient's respiratory symptoms occur only with exercise and given the high degree of effectiveness with SABAs, the NP recommends the prophylactic use of a SABA before undertaking any physical activity.

According to the EPR-3 guidelines, EIB should limit neither participation nor success in vigorous activities. The NP informs the patient that there is no need to avoid recreational activity and, indeed, that with judicious use of her new inhaler she should be able to participate fully in physical activity and may also gain control over her exercise-induced respiratory symptoms. The NP teaches the patient that a warm-up period before exercise may reduce the degree of EIB and that a mask or scarf over the mouth may attenuate cold air-induced EIB (U.S. Department of Health and Human Services, 2007). Increased physical activity may also lead to reduced body mass index and blood pressure. The NP asks the patient to call with an update within 2 weeks and recommends a formal evaluation by an asthma specialist to further assess her for respiratory symptoms.

Peak flow monitoring, after establishing the patient's best baseline peak flow, can be used as needed to monitor patients with symptoms of EIB. All patients, regardless of the presence of asthma, should be encouraged to participate in a conditioning exercise program consisting of moderate to vigorous exercise lasting at least 20 min 3–4 times each week. Exercise recommendations should be complemented by nutrition and dietary monitoring and appropriate weight reduction targets in applicable patients (Haskell et al., 2007).

## Case resolution

The patient returns to her NP 4 weeks later and reports that a follow-up visit with an asthma specialist revealed no underlying asthma. She further reports prevention of respiratory symptoms with use of albuterol pressurized metered dose inhaler before exercise, which she is using no more than twice a week. She reports accepting a job that requires frequent walking between buildings, walking the track at the middle school with her daughters at least twice a week without any respiratory symptoms, and losing 4 lb. She reports feeling more energetic, more positive about her health, and is pleased with her weight loss and control of EIB symptoms.

## Discussion

This case highlights the implications of undermanaged EIB and the possible consequences of decreased physical activity or sedentary lifestyle, which can lead to cardiometabolic disease (Gaesser, Angadi, & Sawyer, 2011; Leiter et al., 2011; McGuire, Janssen, & Ross, 2009; Thorp, Owen, Neuhaus, & Dunstan, 2011). To minimize the risks of obesity and cardiac and metabolic disease in later life, it is important that EIB should not limit participation in physical activity, particularly as effective treatments are readily available. Inhaled SABAs can attenuate EIB in 80%–95% of patients with asthma (Milgrom & Taussig, 1999) and are effective for 2–3 h during exercise; preexercise treatment with a SABA, possibly in combination with long-term controller therapy, is recommended.

Furthermore, Guidelines for the Diagnosis and Management of Asthma (U.S. Department of Health and Human Services, 2007) indicate that patients with EIB should be monitored regularly to ensure their symptoms, including exercise-associated shortness of breath, chest tightness, cough, and wheezing, are well controlled and are not representative of uncontrolled asthma. Patients with persistent symptoms should be evaluated for an appropriate modification of treatment, including addition of a long-term controller medication (U.S. Department of Health and Human Services, 2007). Patients with a compromised level of physical activity because of EIB who do not respond to these conventional treatment strategies should be referred to an asthma specialist for further evaluation and treatment.

## Conclusions

EIB is a well established, easily managed, and often underdiagnosed condition. Patients with EIB should be encouraged to participate fully in physical exercise. Healthcare providers should remain vigilant to identify untreated EIB and ensure that appropriate advice and treatment options are fully discussed with affected patients.

## Disclosures

The authors wish to acknowledge the technical and editorial support provided by ApotheCom with funding from Teva Pharmaceutical Industries, Ltd. Mary Lou Hayden was a consultant for Genentech, Novartis, Teva, Dey Laboratory, and Sunovion, and a member of the speakers' bureau for Merck. Stuart W. Stoloff received grant/research support from and was a consultant for Teva. Gene L. Colice was a consultant and member of the speakers' bureau for Teva, Boehringer Ingelheim, Pfizer, GlaxoSmithKline, Merck, Vatera, and MedImmune. Nancy K. Ostrom received grant/research support from Alexza, Amgen, Astellas, Boehringer Ingelheim, Cephalon, Forest, GlaxoSmithKline, Johnson & Johnson, MAP, MedImmune, Merck, Novartis, Schering-Plough, Sepracor, Sunovian, Teva, and Watson; was a member of the speakers' bureau for AstraZeneca, GlaxoSmithKline, Merck, and Teva; and was a consultant for AstraZeneca, MAP, Novartis, and Teva. Nemr S. Eid was a consultant for Teva and a member of the speakers' bureau for Teva and Merck. Jonathan P. Parsons was a consultant for Teva.
